# Determinants of Early Initiation, Exclusiveness, and Duration of Breastfeeding in Uganda

**Published:** 2014-06

**Authors:** Edward Bbaale

**Affiliations:** School of Economics, Makerere University, Kampala, Uganda

**Keywords:** Breastfeeding duration, Exclusive breastfeeding, Uganda

## Abstract

Breastfeeding practices in Uganda are contrary to the best practice recommended by World Health Organization (WHO). Only six in 10 Ugandan children below the age of six months are exclusively breastfed. This paper investigated the determinants of breastfeeding practices in Uganda. Using the Uganda Demographic and Health Survey (UDHS) of 2006, we employed probit and Cox's regression techniques as well as the Kaplan-Meier survival functions during the analysis. On average, 56% and 46% initiated breastfeeding in the first hour and practised exclusive breastfeeding respectively while 25%, 50%, and 75% terminated breastfeeding at 18, 24, and 26 months respectively. The mean number of months of breastfeeding was 14.1, and the maximum was 40. Hospital delivery increased the probability of early initiation and exclusive breastfeeding by 4-5% (p<0.01) and 7-8% (p<0.01) respectively. Prenatal care increased the probability of early initiation and exclusive breastfeeding by 6-7% (p<0.05) and 5-7% (p<0.05) respectively. Birth intervals less than 24 months increased the risk of early termination of breastfeeding by 19% (p<0.01). Hospital delivery and prenatal care should be made a priority, and mothers should be encouraged to adopt higher birth intervals.

## INTRODUCTION

The role of breastfeeding in ensuring a healthy childhood and survival cannot be underestimated (1-3). Exclusive breastfeeding reduces the risk of childhood illnesses, such as diarrhoea, gastrointestinal and respiratory infections (2-5). Breastfeeding is especially crucial in developing countries where many families cannot afford alternative or supplementary nutrition for their children; hence, it saves household resources (2). Breastfeeding is a natural method of family planning and also promotes sensory and cognitive development (1,6). Indeed, breastfeeding is an important vehicle for the attainment of Millennium Development Goal 4 (to reduce the under-5 mortality rates by two-thirds) within 2015 (7).

To maximize the benefits of breastfeeding, the World Health Organization (WHO) and United Nations Children's Fund (UNICEF) recommend: initiation of breastfeeding within the first hour of life; exclusive breastfeeding (EBF), i.e. the infant only receives breastmilk without any additional food or drink, not even water (apart from syrups and medicines); and breastfeeding on demand, i.e. as often as the child wants, both day and night. The WHO guidelines assert that breastmilk is the natural first food for babies; it provides all the energy and nutrients that the infant needs for the first six months of life; and it continues to provide up to half or more of a child's nutritional needs during the second half of the first year and up to one-third during the second year of life (8,9). WHO recommends colostrum, the yellowish sticky breastmilk produced at the end of pregnancy as the perfect food for the newborn; this can be tapped through early initiation of breastfeeding (8-10).

In line with the WHO guidelines, the Government of Uganda, through the Ministry of Health, is highly committed to enhancing breastfeeding. Through the Ministry of Health, WHO and UNICEF in 1991 launched the Baby-friendly Hospital Initiative (BFHI) in Uganda intended to encourage breastfeeding through education of healthcare workers in maternity and neonatal services. Other initiatives undertaken by the Government through mass media are: education of mothers on how to breastfeed successfully and mobilization of male partners to support breastfeeding mothers. At the community level, peer counsellors have provided support to mothers in a bid to promote exclusive breastfeeding (11).

Despite these efforts, recent statistics for Uganda shade a picture that is contrary to the WHO's recommendations. Only six in 10 Ugandan children below the age of six months are exclusively breastfed. It is no wonder then that the under-five and infant mortality rates stand at 128 and 79 per 1,000 livebirths respectively, which is very high by developing world standards (12). Given these circumstances, there is a growing interest amongst government policy-makers and global health actors in understanding the factors influencing breastfeeding behaviour for informed policy analysis, formulation, and advocacy. Despite this heightened demand, researchers have lent very limited empirical regularity to this subject in the case of Uganda. We found only a few papers (11,13,15-18) addressing breastfeeding issues in Uganda. The major shortcoming of these papers is their concentration on individual parts of the country, casting doubt on the representativeness of their samples and findings. We extend this literature by using a nationally-representative Uganda Demographic and Health Survey of 2006 to answer the following pertinent questions: What factors do influence the duration of breastfeeding? What factors do influence the initiation of breastfeeding in the first hour after birth? What factors do influence exclusive breastfeeding up to six months after birth? Does the type of antenatal care provider matter? Does the place of delivery matter? Answers to these questions should identify the key policy parameters that the government and public health initiatives need to target in order to boost the benefits from breastfeeding.

## MATERIALS AND METHODS

The data were obtained from the Uganda Demographic and Health Survey (UDHS) 2006 conducted by Macro International and Uganda Bureau of Statistics (UBOS) (14). This refers to the UDHS Report (2007) for a complete description of the sampling frame and methods. Ethically, we obtained approval from Macro International to use the UDHS 2006 dataset during the study. Our dependent variables include: (a) initiation of breastfeeding in the first hour after birth—constructed as a discrete binary variable equal to 1 if a mother initiated breastfeeding in the first hour after birth and 0 otherwise; (b) exclusive breastfeeding—constructed as a discrete binary variable equal to 1 if a mother did not feed the baby anything else (with the exception of syrups and medicines) apart from breastmilk for the first six months after birth; (c) duration of breastfeeding—defined as the number of months that the mother reported as having breastfed her baby and is used in our analysis without any further manipulation or construction.

We controlled for various socioeconomic, demographic and behavioural factors. Female education was coded as: 0=no education, 1=primary education, 2=secondary education [those who attained Uganda Certificate of Education (ordinary level) and Uganda Advanced Certificate of Education (advanced level)], 3=post-secondary education (includes vocational and university education). Education of the partner is also coded in the same way. Maternal age was divided into five-year cohorts: 15-19, 20-24, 25-29, 30-34, 35-39, 40-44, and 45-49. The wealth status (an asset index where indicators common to both urban and rural areas are used in creating wealth scores for households in both areas) of the household was divided into five equal groups (quintiles) coded as: 1=poorest, 2=poor, 3=middle, 4=rich, and 5=richest. Religious affiliations were coded as: 1=Catholics, 2=Protestant, 3=Muslim, and 4=‘Others’ which include Evangelicals, Adventists, Orthodox, and Traditionalists. For location, we constructed a dummy variable equal to 1 if a mother dwells in the rural area and 0 if she lives in the urban area. We constructed regional dummy variables: 1=central, 2=east, 3=north, and 4=west. We generated a dummy variable equal to 1 if a mother obtained professional antenatal care and 0 otherwise [According to UBOS and Macro International (2007) professional antenatal care means when women receive antenatal care at least once from a skilled provider (doctor, nurse/midwife, or medical assistant/clinical officer); 94% of women who gave birth in the five years preceding the survey received antenatal care from a skilled provider in Uganda].

Additionally, we generated a dummy variable equal to 1 if a mother delivered in a health facility (hospital, health centre, and clinic) and 0 otherwise.

To estimate the determinants of first-hour initiation and exclusive breastfeeding, we employed the maximum likelihood probit technique, and we generated marginal effects to facilitate easy interpretation. On the other hand, to estimate duration of breastfeeding, we employed the Cox's Proportional Hazards Model. This technique is widely used in the contemporary literature for analysis of durations. The greatest virtue of this method is that it adjusts for truncation bias since our dataset may contain both women who have completed and those who have not completed their breastfeeding episodes by the time the survey was conducted. The hazard rate for failure at time *t* is defined as the rate of failures at time *t* among those who have survived to time *t*. The hazard rate is modelled through the following equation as a function of the baseline hazard (*h_0_*) at time *t* and the effects of one or more χ variables (19):


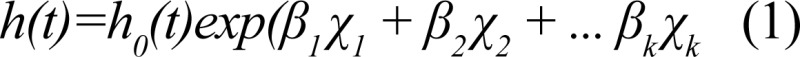


Baseline hazard means the hazard for an observation with all χ variables equal to zero. Cox's regression estimates this hazard rate non-parametrically and obtains maximum likelihood estimates of the β parameters.

## RESULTS

Table 1 presents the descriptive analysis of the initiation of breastfeeding in the first hour and exclusive breastfeeding up to six months after childbirth. It shows that, on average, 56% and 46% of women in Uganda initiated breastfeeding in the first hour after birth and practised exclusive breastfeeding for the first six months respectively.

**Figure 1.**
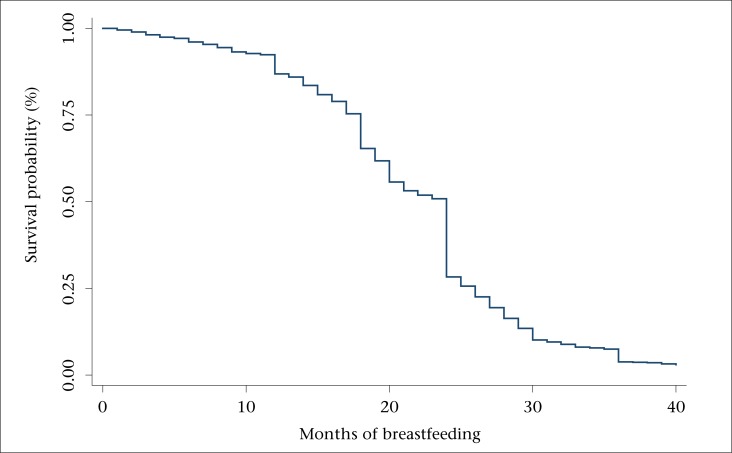
Kaplan-Meier survival function for breastfeeding

**Figure 2.**
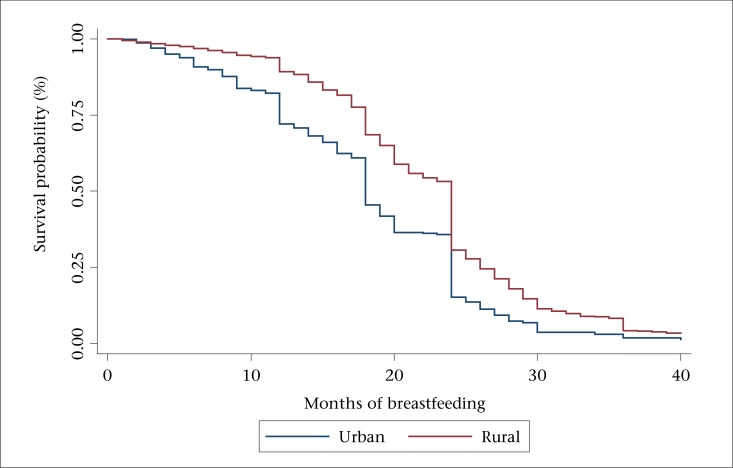
Kaplan-Meier survival function by location

**Figure 3.**
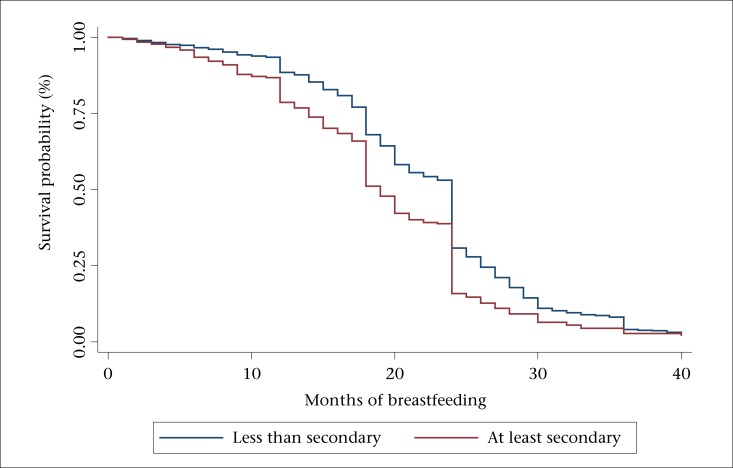
Kaplan-Meier survival function by education

The Kaplan-Meier survival functions (Figure 1 to 3) reveal three outstanding stages at which mothers terminated breastfeeding: 12, 18, and 24 months (Figure 1). The mean number of months of breastfeeding was 14.1, and the maximum reported was 40 months. The Kaplan-Meier survival function for rural women was above that of urban women, indicating that, on average, rural women breastfed for a longer time compared to urban women (Figure 2). Similarly, the survival function for women with less than secondary education was above that of women with post-secondary education, meaning that less-educated women tended to breastfeed for a longer time (Figure 3).

Mothers giving birth in a hospital or clinic were 4-5% (p<0.01) more likely to initiate breastfeeding in the first hour compared to counterparts giving birth at home (Table 2). They were also 7-8% (p<0.01) more likely to practise exclusive breastfeeding compared to counterparts giving birth at home (Table 3). The descriptive findings in Table 1 also confirm these regression results. On the other hand, hospital delivery increased the risk of early termination of breastfeeding by 11% (p<0.1) (Table 4).

Mothers who sought professional antenatal care were 6-7% (p<0.01) more likely to initiate breastfeeding in the first hour compared to counterparts who sought non-professional care (Table 2). Mothers who sought professional care were 5-6% (p<0.05) more likely to exclusively breastfeed their babies compared to their counterparts (Table 3). Table 1 shows that 57% of women who sought professional antenatal care initiated breastfeeding in the first one hour compared to 50% of their counterparts; 47% of women who attended professional antenatal care practised exclusive breastfeeding compared to only 38% of their counterparts.

Mothers in the rural area were 7% (p<0.05) less likely to practise exclusive breastfeeding compared to counterparts in urban areas (Table 3). Being in the rural area reduced the probability of early termination of breastfeeding by 18-23% (p<0.05) compared to their counterparts in urban areas (Table 4); 61% of women in the urban areas initiated breastfeeding in the first hour compared to 56% of women in the rural areas. Additionally, 48% of women in the urban area practised exclusive breastfeeding compared to 46% in the rural area (Table 1). There were significant differences in the breastfeeding practices by region, marital status, and religion (Table 2 and 3).

Mothers in the poor, middle, rich, and the richest wealth quintile were 6-10% (p<0.01) less likely to practise exclusive breastfeeding compared to counterparts in the poorest wealth quintile (Table 3). Table 1 shows that 62% of women in the richest quintile initiated breastfeeding in the first hour compared to only 53% in the poorest quintile. Despite the early initiation, the rich were unlikely to sustain exclusive breastfeeding up to six months; 58% of women in the poorest quintile practised exclusive breastfeeding compared to only 42% in the richest quintile. In the same vein, being in the rich and richest wealth quintile compared to those in the poorest increased the probability of the risk of early termination of breastfeeding by 17-26% (p<0.05) (Table 4).

Our findings also reveal that mother's and father's education was imperative in influencing the duration of breastfeeding (Table 4). Mothers with post-secondary education increased the risk of early termination of breastfeeding by 32% (p<0.1) compared to counterparts with no education. Yet, fathers with primary and secondary education increased the risk of early termination of breastfeeding by 17-18% (p<0.05) compared to counterparts with no education. Table 1 shows that 65% of women with post-secondary education initiated breastfeeding in the first hour compared to only 52% with no education. However, 50% of women with no education practised exclusive breastfeeding compared to only 46% with post-secondary education. The same pattern can be observed for partner's education for both early initiation and exclusive breastfeeding. Along the same line of argument, mothers in the agricultural sector reduced the risk of early termination of breastfeeding by 17% (p<0.1) compared to counterparts having white collar jobs.

**Table 1.** Average percentages of women who initiated breastfeeding in the first hour after birth and those who practised exclusive breastfeeding in the last five years prior to the 2006 UDHS by background characteristics in Uganda

Characteristics	Breastfeeding in the 1st hour	Exclusive breastfeeding in the first 6 months
Antenatal care: Professional	57.3	47
Non-professional	50	38
Place of antenatal care: Home	55	38.5
Hospital/clinic	57	46.1
Place of delivery: Home	54	45.2
Hospital/clinic	59.1	47
Mother's education: No education	52	50
Primary	57	45
Secondary	56.4	44
Post-secondary	64.5	46
Partner's education: No education	51.5	46
Primary	56	46
Secondary	60	46
Post-secondary	60	44
Location: Urban	60.5	47.5
Rural	55.5	45.5
Region: Central	58.1	39
East	57	43.2
North	50.4	60.4
West	60	42.2
Wealth quintile: Poorest	53	58
Poorer	55.3	47.5
Middle	55	39.1
Rich	57	41
Richest	62	42.2
Birth interval: Less or equal to 24 months	59.2	45.7
More than 24 months	57	46.3
Mother's occupation: White collar job	60	41.4
Agriculture	55.2	47
Services	62	45.4
Blue collar job	56.2	44.1
Partner's occupation: White collar job	62	42.5
Agriculture	55	48
Services	59.3	33.2
Blue collar job	56.4	45.4
Using infant bottle + nipple: Yes	−	40.1
No	−	47.2
Married: Yes	57	47
No	53	42
Child's gender: Male	55	45
Female	58	46.3
Child's age: 0-2 month(s)		47
3-5		42
Total	56	46

Source: Authors’ own calculations from UDHS 2006

Surprisingly, the gender of the baby matters for the duration of breastfeeding. Being a male baby increased the risk of early termination of breastfeeding by 12-16% (p<0.01) compared to female counterparts (Table 4). Table 1 shows that mothers were more likely to initiate breastfeeding to female babies in the first hour and to exclusively breastfeed them compared to male babies. Birth intervals did matter. Mothers whose birth intervals were less than 24 months increased the risk of early termination of breastfeeding by 19% (p<0.01) compared to counterparts with at least 24 months birth interval (Table 4). Table 1 shows a high percentage of mothers (59%) with birth interval less than 24 months, who initiated breastfeeding in the first hour compared to those (57%) with birth interval of at least 24 months.

## DISCUSSION

The study set out to investigate the factors associated with the initiation, exclusiveness, and duration of breastfeeding in Uganda. The average percentages of women who initiated breastfeeding in the first hour and those who practised exclusive breastfeeding were very low, given the WHO guidelines. In the same line of argument, the average percentages of women initiating breastfeeding in the first hour and those practising exclusive breastfeeding in the different regions were quite low, and the central region was at an extreme disadvantage. These results imply that mothers in other regions breastfed longer compared to mothers in the central region. This can be attributed to the fact that the central region was more urban than other regions and, hence, mothers’ economic activities were more likely to conflict with their breastfeeding practices, which led to early termination. The differences observed by residence in relation to the duration of breastfeeding can be attributed to the nature of economic activities undertaken by rural dwellers compared to those of their counterparts, which, in most cases, did not conflict with their maternal roles.

**Table 2.** Determinants of initiation of breastfeeding in the first hour after birth

Variable	Model 1	Model 2	Model 3
Hospital delivery	0.0397**		0.0453***
	(0.0174)		(0.00849)
Professional antenatal care	0.0604**	0.0605**	0.0701***
	(0.0141)	(0.0139)	(0.00614)
Mother's education: None	0.0169	0.0184	
	(0.381)	(0.337)	
Primary	−0.00325	0.000515	
	(0.917)	(0.987)	
Secondary	0.0518	0.0556	
	(0.381)	(0.346)	
Location: Rural	−0.0101	−0.0115	0.00759
	(0.727)	(0.691)	(0.824)
Region: East	−0.00250	−0.00170	−0.0147
	(0.917)	(0.944)	(0.567)
North	−0.0587**	−0.0597**	−0.0731***
	(0.0221)	(0.0198)	(0.00681)
West	0.0421*	0.0406*	0.0277
	(0.0839)	(0.0957)	(0.288)
Married	0.0379*	0.0375*	0.0379
	(0.0923)	(0.0955)	(0.105)
Religion: Protestant	−0.0431**	−0.0434**	−0.0375**
	(0.0142)	(0.0135)	(0.0411)
Muslim	0.0306	0.0305	0.00432
	(0.234)	(0.236)	(0.876)
Other	−0.0428	−0.0438*	−0.0451
	(0.106)	(0.0982)	(0.104)
Professional birth attendant		−0.0271	
		(0.111)	
Observations	4,583	4,584	4,202

Robust p values in parentheses;

***p<0.01;

**p<0.05;

*p<0.1;

In all the three models, we controlled for the wealth status, education of the father, and child's sex. In Model 3, we controlled for occupation of the mother and father. The results have not been displayed to save space because these are insignificant

Consequently, mothers in rural areas had longer episodes of breastfeeding compared to those living in the urban areas. This corroborates our finding shown in Figure 2 of the Kaplan-Meier survival function and the previous authors (1,20-22). The importance of companionship in breeding a healthy baby is also underlined in our findings. Married women were more likely to follow proper breastfeeding practices just as the previous authors had found (13). Therefore, mass mobilization of male partners to support breastfeeding mothers is a good strategy that can help entrench best practices of breastfeeding in the country.

**Table 3.** Determinants of exclusive breastfeeding in the first six months after birth

Variable	Model 1	Model 2	Model 3
Hospital delivery	0.0736***		0.0774***
	(3.24e-05)		(2.16e-05)
Professional antenatal care	0.0565**	0.0535**	0.0650**
	(0.0276)	(0.0366)	(0.0151)
Mother's education: Primary	-0.0191	-0.0189	
	(0.348)	(0.351)	
Secondary	0.00242	0.000595	
	(0.942)	(0.986)	
Post-secondary	-0.0311	-0.0357	
	(0.609)	(0.558)	
Location: Rural	-0.0474	-0.0446	-0.0724**
	(0.120)	(0.144)	(0.0474)
Region: East	-0.0199	-0.0162	-0.0131
	(0.435)	(0.523)	(0.631)
North	0.138***	0.141***	0.153***
	(3.67e-07)	(1.68e-07)	(6.02e-08)
West	0.0356	0.0378	0.0410
	(0.167)	(0.143)	(0.136)
Married	0.0488**	0.0478**	0.0472*
	(0.0433)	(0.0473)	(0.0599)
Religion: Protestant	-0.0641***	-0.0655***	-0.0721***
	(0.000498)	(0.000371)	(0.000169)
Muslim	-0.0940***	-0.0969***	-0.112***
	(0.000472)	(0.000309)	(0.000111)
Other	-0.0495*	-0.0537*	-0.0648**
	(0.0717)	(0.0505)	(0.0236)
Wealth quintile: Poor	-0.0284	-0.0371	-0.0298
	(0.242)	(0.117)	(0.211)
Middle	-0.0988***	-0.108***	-0.0943***
	(0.000279)	(4.41e-05)	(0.000468)
Rich	-0.0671**	-0.0846***	-0.0590**
	(0.0223)	(0.00261)	(0.0409)
Richest	-0.0843**	-0.103***	-0.0647*
	(0.0192)	(0.00300)	(0.0795)
Feeding with bottle	-0.0391*	-0.0413*	-0.0249
	(0.0708)	(0.0559)	(0.285)
Professional birth attendant		-0.0786***	
		(1.09e-05)	
Observations	4,259	4,263	3,907

Robust p values in parentheses;

***p<0.01;

**p<0.05;

*p<0.1;

In all the three models, we controlled for education of the father and child's gender. In Model 3, we controlled for occupation of the mother and father. The results have not been displayed to save space because these are insignificant

**Table 4.** Determinants of breastfeeding duration (probability of risk occurrence after Cox's PH regression)

Variable	Model 1	Model 2	Model 3
Hospital delivery	0.111*	0.0949	0.0828
	(0.0521)	(0.110)	(0.180)
Professional antenatal care	−0.0737	−0.0388	−0.0633
	(0.520)	(0.747)	(0.606)
Location: Rural	−0.179**	−0.177*	−0.234**
	(0.0389)	(0.0749)	(0.0121)
Region: East	−0.175**	−0.180**	−0.148*
	(0.0248)	(0.0268)	(0.0739)
North	−0.718***	−0.785***	−0.708***
	(0)	(0)	(0)
West	−0.350***	−0.349***	−0.342***
	(1.12e-05)	(3.55e-05)	(6.48e-05)
Married	−0.189***	−0.182***	−0.254***
	(0.00465)	(0.00878)	(0.000530)
Child's gender: Male	0.159***	0.131**	0.116**
	(0.00159)	(0.0130)	(0.0324)
Child's age: 3-5 months	−0.119**	−0.144**	−0.105*
	(0.0307)	(0.0131)	(0.0743)
Wealth quintile: Poor	0.0881	0.0918	0.0893
	(0.295)	(0.281)	(0.320)
Middle	0.0221	0.0143	−0.00642
	(0.809)	(0.878)	(0.947)
Rich	0.169*	0.113	0.176*
	(0.0711)	(0.239)	(0.0766)
Richest	0.222**	0.119	0.265**
	(0.0460)	(0.298)	(0.0264)
Partner's education: Primary	0.167**		0.177**
	(0.0388)		(0.0383)
Secondary	0.167*		0.159
	(0.0742)		(0.113)
Post-secondary	−0.0594		−0.0392
	(0.647)		(0.779)
Mothers education: Primary	−0.00367		−0.00265
	(0.958)		(0.971)
Secondary	0.0164		−0.0280
	(0.875)		(0.806)
Post-secondary	0.318*		0.209
	(0.0764)		(0.313)
Mother's occupation: Agriculture		−0.166*	
		(0.0670)	
Services		0.174	
		(0.192)	
Blue collar job		−0.200*	
		(0.0997)	
Birth interval: Less than 24 months			0.191***
			(0.00140)
Observations	4,061	3,719	3,513

Robust p values in parentheses;

***p<0.01;

**p<0.05;

*p<0.1;

In all the three models, we controlled for religion. In Model 3, we controlled for occupation of the father. The results have not been displayed to save space because these are insignificant

Just as in Dearden *et al*. (5), hospital delivery is revealed important in igniting favourable breastfeeding practices. This can be attributed to the quality of information that mothers receive from hospitals or clinics compared to the information obtained from home (mainly from traditional birth attendants). Therefore, the government policy that can create incentives for mothers to give birth in hospitals is called for. Astonishingly, hospital delivery was associated with early termination of breastfeeding. This can probably be attributed to a small proportion of mothers delivering at the health facilities and those delivering with assistance of a skilled care provider [41% and 42% respectively (14)]. Thus, despite the existence of initiatives in hospitals and health centres, like the Baby-friendly Hospital Initiative, a large proportion of women were outside the net of these initiatives. Besides, these initiatives were more concentrated in the urban compared to rural health centres. This is the reason why Table 1 indicates that a large proportion of women in the urban area initiated breastfeeding in the first hour and also practised exclusive breastfeeding compared to their rural counterparts.

Professional antenatal care was associated with early initiation and exclusive breastfeeding. Previous authors also found antenatal care to be significant in influencing breastfeeding (1,16). This can be attributed to the information that mothers obtained from antenatal counselling sessions concerning the importance of early initiation and exclusive breastfeeding. Therefore, the government policy intended to encourage more mothers to seek professional antenatal care is very paramount. Such efforts as outreach antenatal clinics staffed with professional workers will go a long way in achieving this objective. However, professional antenatal care was not associated with the duration of breastfeeding probably because mothers lost contact with health workers after birth and might forget the importance of prolonged breastfeeding. It is noteworthy that some authors did not find prenatal care to be significant in influencing early initiation and exclusive breastfeeding (3,23).

The rich were less likely to practise exclusive breastfeeding. This can be attributed to their ability to pay for supplementary foodstuffs, like infant formula that can be given to their children in addition to breastmilk. Yet, the poorest might not afford the supplementary foods and, hence, might rely on exclusive breastfeeding, which is good up to six months when a baby can be fed complementary foods. Previous authors also found wealth status to be importantly associated with breastfeeding behaviour (2,3,10,23). The government policy should make sure that exclusive breastfeeding is not pegged to the ability to pay, rather on doing the right thing at the right time. The poorest should also be able to feed their children appropriate supplementary foods, starting with the sixth month; failure to do so due to inability to pay may lead to stunted growth of children.

Educated mothers and those in formal employment had a disadvantage over their counterparts in the breastfeeding practices. Previous authors also found type of occupation (1,2,3,5,10,20) and maternal education (1,2,23,24) to be imperatively associated with breastfeeding practices. Higher education increased the probability that mothers working outside their homes could conflict with the breastfeeding practices. On the other hand, agricultural activities were normally near home and were non-formal, making it possible for mothers to breastfeed their children in longer episodes. The Government should come up with a new labour law that allows mothers with young children a longer leave and also to have an interval in between work to breastfeed. The law may allow them to reach work an hour later or leave work an hour earlier than others for the first six months after childbirth.

According to the Uganda Employment Act of 2006 (25) “a female employee shall, as a consequence of pregnancy, have the right to a period of sixty working days leave on full wages of which at least four weeks shall follow childbirth or miscarriage.” Whereas this looks fair, it does not apply universally across the public and private sectors, with women working in the private sector being at an extreme disadvantage. Employers in the private sector do not always allow mothers to enjoy the full length of the maternity leave and, hence, their children can only be breastfed exclusively for a very short period of time not exceeding two months. Whereas only 3% of women, according to UBOS and Macro International (14), are engaged in white collar jobs, the problem of lack of breastfeeding time for infants extends beyond this to every woman who is engaged in paid employment. Overall, maternity leave legislation needs to be made more effective in the private sector, along with adding more days of leave.

Religion is used to capture the behaviour, traits, or cultural beliefs of women and is significantly associated with breastfeeding practices (13). The Government should urge the respective religious leaders to ‘preach’ to their congregations the benefits of breastfeeding to the mother and her child and that exclusive and early initiation of breastfeeding are especially encouraged. This appears plausible in Uganda which has three deeply-entrenched religious denominations (Catholics, Protestants, and Muslims), with very many followers across the different sections of the population. Additionally, the new sects, such as Born-again Christians and Adventists, have attracted multitudes of followers. Hence, if the best practices of breastfeeding are articulated at different places of worship, one would expect a greater response.

### Strengths and limitations

The greatest strength of this paper is its use of data from a nationally-representative survey, which enhances generalization of results for the entire country. However, the primary source of limitation is the recall bias. Data were called retrospectively for the past five years and, hence, mothers might not have been in position to recall correctly all the events that took place from the time of breastfeeding initiation to termination.

### Conclusions

This paper set out to investigate the determinants of early initiation, exclusiveness, and the duration of breastfeeding in Uganda. We employed the maximum likelihood probit and Cox's regression techniques as well as the Kaplan-Meier survival functions in the analysis. Our results reveal that hospital delivery, professional antenatal care, education of parents, occupation of mothers, birth intervals, child's gender, wealth status, location, and regional differences were significant factors associated with breastfeeding practices in Uganda.

Our findings yielded interesting policy messages. To encourage early initiation, exclusive and longer breastfeeding, mothers should be encouraged to book for childbirth in hospitals where they interface with professionals who can advise and assist in the process of breastfeeding to commence. Earlier than that, mothers should be given incentives to seek professional antenatal care where they are given counselling on the importance of early initiation and exclusive breastfeeding and other issues relating to child nutrition. The Government should also establish an outreach programme that can create community-level or village-level clinics and health centres that will make it easier for mothers to seek professional care and hospital delivery. The location and regional disparities observed may partly be due to skewed information flow. The Government should package a standard piece of information on breastfeeding that can be disseminated in all parts of the country, using media and outreach seminars at village level as well as at health centres. Mothers working outside homes, in formal public and private institutions, should be protected by a relevant labour law that makes their work friendly to breastfeeding.

Future research should endeavour to ascertain the relationship between breastfeeding practices, child's nutritional status and under-five mortality.

## ACKNOWLEDGEMENTS

I wish to acknowledge the Centre for Global Development (CGD) for giving me the opportunity as a Visiting Fellow that allowed me the time to write this article. I also thank Makerere University for granting me leave and IDRC for sponsoring the programme. However, the views expressed in this article are those of the author and not of any of the institutions mentioned.

## References

[1] Abada TSJ, Trovato F, Lalu N (2001). Determinants of breastfeeding in the Philippines: a survival analysis. Soc Sci Med.

[2] Islam S, Yadava KNS, Alam MA (2006). Differentials and determinants of the duration of breastfeeding in Bangladesh: a multilevel analysis. Proc Pakistan Acad Sci.

[3] Chudasama RK, Patel PC, Kavishwar AB (2009). Determinants of exclusive breastfeeding in South Gujarat region of India. J Clin Med Res.

[4] Yoon PW, Black RE, Moulton LH, Becker S (1996). Effect of not breastfeeding on the risk of diarrheal and respiratory mortality in children under 2 years of age in Metro Cebu, The Philippines. Am J Epidemiol.

[5] Dearden K, Altaye M, De Maz I, De Oliva M, Stone-Jimenez M, Morrow AL (2002). Determinants of optimal breast-feeding in peri-urban Guatemala City, Guatemala. Rev Panam Salud Publica.

[6] Guz D, Habcraft J (1991). Breastfeeding and fertility: a comparative analysis. Popul Stud.

[7] Jones G, Steketee RW, Black RE, Bhutta ZA, Morris SS (2003). the Bellagio Child Survival Study Group. How many children deaths can we prevent this year?. Lancet.

[8] Butte NF, Lopez-Alarcon MG, Garza C (2002). Nutrient adequacy of exclusive breastfeeding for the term infant during the first six months of life.

[9] Kramer MS, Kakuma R (2002). The optimal duration of exclusive breastfeeding: a systematic review.

[10] Haroun HM, Mahfouz MS, Ibrahim BY (2008). Breast feeding indicators in Sudan: a case study of Wad Medani town. Sudanese J Public Health.

[11] Tylleskär T, Jackson D, Meda N, Engebretsen IM, Chopra M, Diallo AH (2011). PROMISE-EBF Study Group. Exclusive breastfeeding promotion by peer counsellors in sub-Saharan Africa (PROMISE-EBF): a cluster-randomised trial. Lancet.

[12] US Agency for International Development. MNPI: Maternal and Neonatal Program Effort Index. 2006. (http://www.policyproject.com/pubs/mnpi.cfm, accessed on 17 December 2012).

[13] Uganda Bureau of Statistics. (2007). Uganda demographic and health survey 2006.

[14] Engebretsen IMS, Wamani H, Karamagi C, Semiyaga N, Tumwine J, Tylleskär T (2007). Low adherence to exclusive breastfeeding in Eastern Uganda: a community-based cross-sectional study comparing dietary recall since birth with 24-hour recall. BMC Pediat.

[15] Wamani H, Astrøm AN, Peterson S, Tylleskär T, Tumwine JK (2005). Infant and young child feeding in western Uganda: knowledge, practices and socio-economic correlates. J Trop Pediatr.

[16] Ssenyonga R, Muwonge R, Nankya I (2004). Towards a better understanding of exclusive breastfeeding in the Era of HIV/AIDS: a study of prevalence and factors associated with exclusive breastfeeding from birth, in Rakai, Uganda. J Trop Pediatr.

[17] Pool R, Nyanzi S, Whitworth JA (2001). Breastfeeding practices and attitudes relevant to the vertical transmission of HIV in rural south-west Uganda. Ann Trop Paediatr.

[18] Nankunda J, Tumwine JK, Soltvedt A, Semiyaga N, Ndeezi G, Tylleskär T (2006). Community based peer counsellors for support of exclusive breastfeeding: experiences from rural Uganda. Int Breastfeed J.

[19] Hamilton LC Statistics with Stata: version 10. Belmont: Brooks/Cole 2009, 490 p.

[20] Huffman SL (1984). Determinants of breastfeeding in developing countries: overview and policy implications. Stud Fam Plann.

[21] Akin JS, Bilsborrow RE, Guilkey DK, Popkin BM (1986). Breastfeeding patterns and determinants in the near east: an analysis for four countries. Popul Stud.

[22] Ferry B, Smith DP (1983). Breastfeeding differentials.

[23] Alemayehu T, Haidar J, Habte D (2009). Determinants of exclusive breastfeeding practices in Ethiopia. Ethiopian J Health Dev.

[24] Caldwell JC (1979). Education as a factor in mortality decline: an examination of Nigerian data. Popul Stud.

[25] Uganda. Ministry of Public Service. The Employment Act, 2006. Kampala: LDC Publishers, 2006. 54 p. (http://www.mglsd.go.ug/wp-content/uploads/2013/07/Laws/employment%20Act%202006.pdf, accessed on 21 December 2012).

